# Effects of Intestinal Flora on Irritable Bowel Syndrome and Therapeutic Significance of Polysaccharides

**DOI:** 10.3389/fnut.2022.810453

**Published:** 2022-05-11

**Authors:** Yang Ye, Yanan Liu, Kejun Cheng, Zufang Wu, Peng Zhang, Xin Zhang

**Affiliations:** ^1^Department of Food Science and Engineering, Ningbo University, Ningbo, China; ^2^Chemical Biology Center, Lishui Institute of Agriculture and Forestry Sciences, Lishui, China; ^3^Department of Student Affairs, Xinyang Normal University, Xinyang, China

**Keywords:** intestinal flora, IBS, GBA, polysaccharides, short chain fatty acids

## Abstract

In recent years, the relevant research on intestinal flora has been in full swing, and it has become an extremely important research direction in clinical medicine and life science. Irritable bowel syndrome (IBS) is a common disease characterized by changes in intestinal function and accompanied by comorbid anxiety. At present, the pathogenic mechanism of IBS is not yet clear. The gut-brain axis (GBA), as a two-way information exchange system between the gut and the brain, has an important influence on the prevention of IBS. Present studies have shown that polysaccharides are important for maintaining the steady status of intestinal micro-environment. This review summarized the relationship between intestinal flora, GBA and immune activation, and provided a new idea for the preventive treatment of IBS from the perspective of polysaccharides.

## Introduction

The gut microbiota is a source of the genetic and metabolic diversity of humans (1). With the development of the times, people's knowledge of the types, characteristics and models of action of the gut microbiota has grown rapidly (2). The human intestinal flora consists of at least 10^13^ species of microorganisms. Their collective genomes contain 100 times as many genes as ours, creating complex and body-specific adaptive ecosystems that are perfectly adapted to physiological changes of the host (3, 4). The intestinal flora is a complex ecological community that affects normal physiology and disease susceptibility through their collective metabolic activities and host interactions (5). Homeostasis, that is, the human body controls its internal environment to maintain relative stable, so that the body restore to a relatively balanced state. The stabilization of the gut microbiota is of great significance to the homeostasis (6). As an important part of the human body, the intestinal flora plays a considerable role in antibacterial, immune and metabolism (7, 8). Resident bacteria in the gut can prevent external pathogens from entering the gut mucosa, and symbiotic microorganisms can also use surrounding substances to maintain gastrointestinal homeostasis. For instance, dietary nutrients are absorbed by the intestine and co-metabolized by host enzymes along with various non-nutritive compounds produced by the microbiota (6, 9). The disturbance in the homeostasis of the intestinal flora can damage the intestinal mucosal barrier and cause declined immune function (10). The disorder of gut flora is associated with many diseases, including IBS, depression, multiple sclerosis, diabetes, autism and cancer (11).

The integrity of intestinal barrier mainly comes from the balanced crosstalk between intestinal flora, mucus, enterocytes, the immune system, and the intestinal vascular barrier, involving network, bidirectional crosstalk, and inflammation control. It is of vital significance in maintaining intestinal homeostasis (12). These intricate interactions significantly affect other organs and physiological functions (13). The intestinal flora can alter nutrient absorption, metabolite release, and digestive system permeability, and regulate bone growth through the gut-bone axis. Studies have shown that increasing the number of *Bifidobacteria* in the gastrointestinal tract can alter the absorption levels of minerals such as magnesium and calcium, thereby increasing bone density (14). Typically, patients with inflammatory bowel disease (IBD) are at increased risk for coronary heart disease, suggesting a two-way communication network through the gut-heart axis (15). Similarly, a fat-rich diet can alter the gut microbiota and gut vascular barrier through the gut-liver axis, allowing bacterial products in the portal vein to flow to the liver, thereby affecting systemic inflammation (16).

In general, the interaction between the intestinal flora and the brain mainly indirectly affect cognition, sleep, learning ability, and emotions through the nervous, endocrine, immune, and metabolic systems. This two-way information exchange system that integrates brain and intestinal functions is called the gut-brain axis (17). Irritable bowel syndrome (IBS) is a common disease characterized by changes in intestinal function and accompanied by comorbid anxiety (18). The health impact of the disease is similar to depression, resulting in a significant economic burden (19). At present, the pathogenic mechanism of IBS is not clear. Studies have shown that genetic and environmental factors, immune response, and psychological factors such as depression and anxiety can affect IBS (20). Changes in bidirectional GBA interactions are believed to have an impact on many intestinal diseases. The two-way communication effect of GBA has been explored and applied to the treatment of various diseases, including depression, IBS, Alzheimer's disease, and so on (21).

Polysaccharides are biopolymers consisting of monosaccharides. To date, more than 300 natural polysaccharides have been identified (22). Polysaccharides can be used to treat functional constipation and constipation-predominant IBS (IBS-C) without significant side effects (23). The intestinal flora can establish a close relationship between the human body and polysaccharides, and the intestinal microenvironment is affected by the gut microorganisms and releases sugars or short-chain fatty acids (SCFAs) that are easily assimilated by the host, and further participating in a series of metabolic reactions in the body (24). At the same time, polysaccharides are involved in regulating the composition of intestinal flora, cytokines, intestinal mucosal repair, which are of great significance to the normal development of the human mucosa immune system (25). This article reviewed the mechanism of intestinal flora on IBS through the enteric nervous system (ENS), central nervous system (CNS) and the immune response of GBA, which provided a brand-new direction for treating IBS from the polysaccharide perspective.

## The Important Role of Intestinal Flora

### The Intestinal Flora

Microorganisms in the human body are able to colonize all exposed surfaces, especially the intestinal surface (26). The human intestinal flora is complex and consists of at least 1,000 different bacteria (27). Gut microbes are estimated to comprise ~0.3% of the body's weight (28). The neonatal gut is sterile, derived primarily from the mother and external environment, and mainly affected by the type of delivery and feeding methods (29). The diversity of neonatal gut microorganisms is low, mainly *Proteobacteria* and *Actinobacteria*. At the age of 2–5, the composition of microorganisms is similar to that of the adult and becomes more diverse (30). The intestinal flora is the main regulator of the immune system, which provides a core set of immune stimulatory molecules to establish symbiotic relationships with the host, promoting the host's metabolism, tissue development and immune maturation, and protecting it from intestinal pathogens (31, 32). A consensus has been reached that when intestinal flora and intestines coexist harmoniously, the human body is healthy (33, 34).

### Changes in Patients With IBS

IBS is a functional gastrointestinal disorder that is easy to see in life, accompanied by abdominal pain, which is related to changes in bowel habits. It is one of the two most common gastrointestinal (GI) disorders, which affects 7–21% of the world's population (18, 35). Due to the different symptoms, IBS patients can be divided into IBS-D with diarrhea as the main symptoms, IBS-C with constipation as the main symptoms, and IBS-M with mixed symptoms (36). Bloating is the most prevalent symptom reported by 96% of IBS patients (37). There are many potential causes of IBS, including changes in the composition of the intestinal flora (38). The overgrowth of small intestinal bacteria is characterized by an abnormally high number of small intestinal bacteria, which has been considered an important feature of patient subgroups (39). In addition, the severity of anxiety, IBS subtypes, and IBS symptoms are associated with the composition of gut bacteria (38). The diversity of potentially pathogenic bacteria in fecal samples of IBS patients increased, while the diversity of the beneficial bacteria decreased (39). Compared with healthy people, the number of *Bacteroidetes* and *Actinobacteria* in the intestines of patients with IBS patients is reduced (40).

## The Role of the Intestinal Flora in IBS

### The Impact on GBA

**Figure 1:**
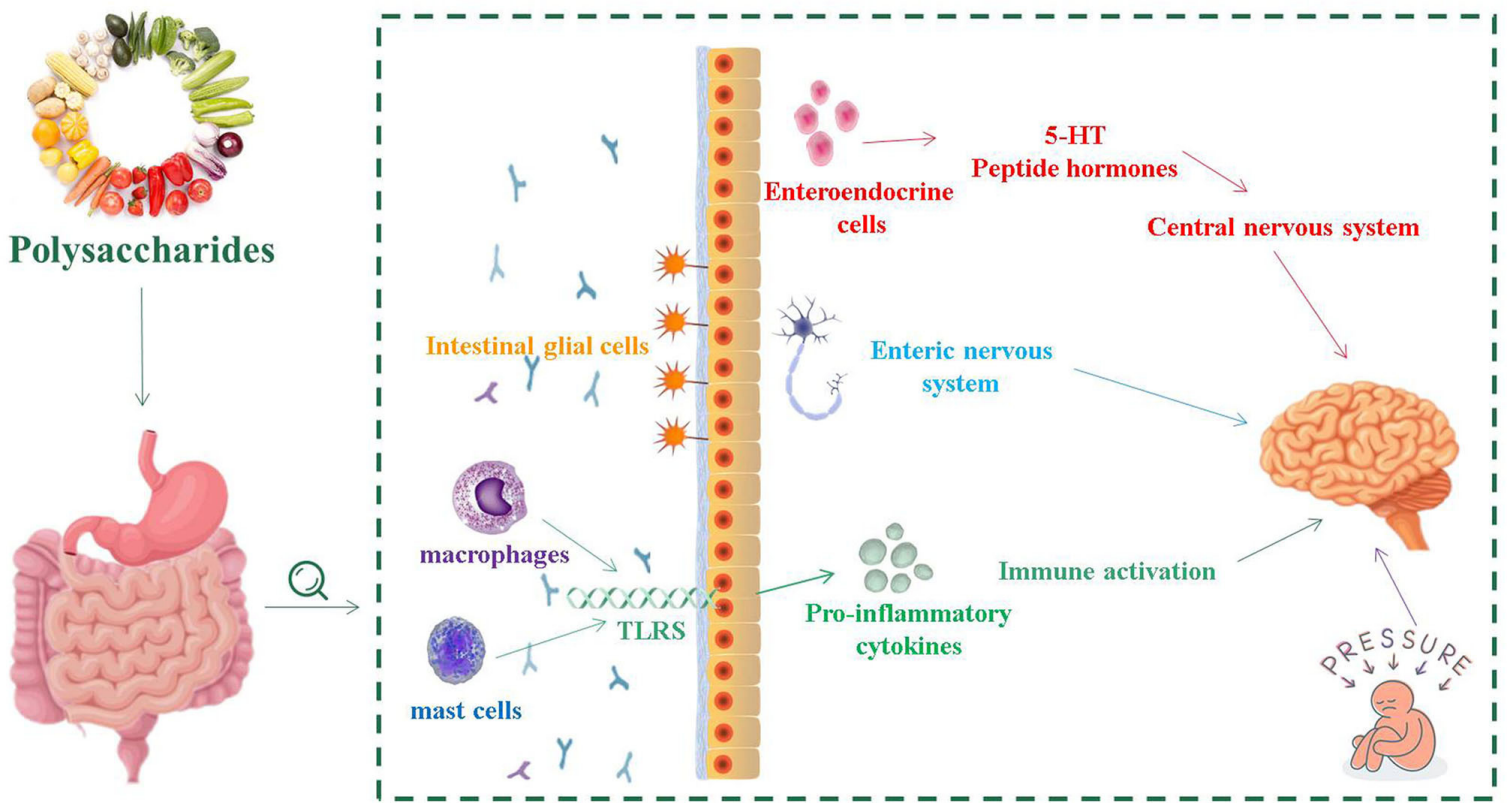
The relationship between intestinal flora and intestinal nervous system, central nervous system and immune activation.

GBA consists of a bidirectional response system that works through neural, endocrine and immune pathways between the brain and the emotional cognitive center in the peripheral gut, including the autonomic nervous system (ANS), ENS and CNS (41). Its role is to monitor and integrate intestinal function, providing a bridge between mood, memory, cognitive function and the gastrointestinal tract, producing multiple effects, such as changing the intestinal permeability and causing immune activation (Figure 1) (42). The consumption of fermented dairy products containing *Bifidobacterium, Lactobacillus* and *Streptococcus thermophilus* can alter brain connections and capabilities, supporting the effects of intestinal microorganisms on GBA (43). The study of germ-free (GF) animals found that the cultivation of intestinal bacteria is central to the maturation of ENS and CNS (44). In recent years, researched showed that patients with IBS usually have micro-ecological disorders, suggesting that IBS is closely related to GBA (45, 46).

### The Impact on ENS

The normal gastrointestinal function requires the involvement of ENS, which is the greatest and most complex part of the peripheral nervous system. ENS includes sensory neurons, interneurons, motor neurons, as well as enteric glia, and controls almost all aspects of gastrointestinal physiology (47, 48). In GF mice, the excitability of post-intestinal hyperpolarized (AH) neurons was reduced and recovered after implantation of the normal intestinal microbiome (49). Enteric glial cells (EGCs) are an important part of ENS, and are associated with neuronal synapses between submucosa neurons and muscles (50). EGCs coordinate myenteric signaling between cells and epithelial cells, regulating intestinal dynamics as well as the secretory and absorption function of the upper intestine (50). The density of EGCs in the intestinal mucosa of sterile mice was significantly decreased, which indicated that the intestinal flora had a remarkable impact on the initial implantation of EGCs in the intestinal mucosa (51). At the same time, EGCs are necessary for the structural and functional integrity of ENS to participate in the intestinal mucosa barrier and promote intestinal stability (52).

### The Impact on CNS

During the development of CNS, the production of neurons is affected by various environmental factors. Gut microbiota played an important role in regulating and guiding the neurodevelopment of CNS. This process primarily occurs in the hippocampus (53). Through the mother-infant interactions, regulators in the gut microorganism activate signaling receptors that develop the fetal nervous system with implications for the formation of acquired learning, memory, cognitive and behavioral responses (54). The vagal nerve (VN) is a mixed nerve containing motor, sensory and parasympathetic nerve fibers. The communication between the microbiota and the brain involves the VN, which transmits information from the lumen environment to CNS (42). Neurological disturbances or neurasthenia can lead to CNS dysfunction, such as mood disorders, neurodegenerative diseases, and gastrointestinal pathology. The ingestion of lactic acid bacteria improves anxiety in mice, which weaken or even disappeared after VN is cut off (55). During the development of CNS, serotonin is crucial in regulating neuronal differentiation and migration, axonal growth, myelin formation and synapse formation, as well as a key neurotransmitter involved in the regulation of mood control, food intake, sleep and pain management (56, 57). The synthesis of serotonin depends on the availability of tryptophan, which is a key monoamine neurotransmitter involved in regulating CNS transmission (58). The complex symbiotic microorganisms living in the gastrointestinal tract of mammalian are the driving forces influencing the metabolism of tryptophan in the gut (59). The main carriers of neurotransmitters that interact with the CNS, such as serotonin, are enteroendocrine cells (EECs). EECs are located in the mucosa of the gastrointestinal tract, which contain at least 20 signaling molecules (60). When stimulated, these signaling molecules respond and are released to the nervous system as well as brain (61). EECs can secrete a variety of peptide hormones and bioactive amines, including 5-hydroxytryptamine (5-HT), which are important for maintaining intestinal homeostasis (62). Chlamydia LPS is present in patients with IBS, and that chlamydia antigens are closely related to EES, confirming that EECs is associated with intestinal infections (63).

### The Activation of Immune Response

The mammalian gut is a complex ecosystem that interacts with three important components: the epithelial and its neuronal connections, the intestinal-related immune tissue, and the symbiotic microbiome (64). The intestinal mucosal barrier separates the external environment from the human environment. In the early development of life, intestinal microorganisms are of great significance to the maturation of the mucosal immune system (11). Inflammatory cells on the surface of the intestinal mucosa of IBS patients, such as mast cells and macrophages, have a low degree of the infiltration (65, 66). Mast cells play various functions, such as regulating the permeability of intestinal epithelial cells, controlling blood coagulation, and promoting smooth muscle peristalsis (67). They can further perform several different roles by releasing pre-formed media (such as proteases, histamine and heparin) from their granules or by releasing recombinant media (including lipid media and cytokines) (67).

Mast cells activate innate immune cells such as toll-like receptors (TLRs) to identify receptors, induce the secretion of anti-inflammatory mediators, and help maintain intestinal immune tolerance (68). Bacterial components in the gut, such as lipopolysaccharides (LPS) in the cell wall components of Gram-negative bacteria, can be acted as ligands for TLRs, and flagellin can also be recognized by TLR5 (69). TLR2 and TLR4 are two signaling receptors that are required for activation by bacterial wall components in TLRs and significantly increase the expression of TLR2 and TLR4 antigens in inflammatory mucosal cells (70). The level of TLR4 in colonic tissues of patients with IBS was remarkably higher than that of healthy controls, indicating that there was a certain degree of immune disorder in IBS patients (71). In addition, increased TLR expression in IBS-M patients induces intracellular signaling pathways, which result in increased expression of the mucosal pro-inflammatory cytokines (72).

## The Effect of Polysaccharides on Intestinal Flora

Polysaccharides are a kind of active natural macromolecular substance, which are composed of glycoside bonds. Polysaccharides are polymeric-polymer carbohydrates composed of at least 10 monosaccharides, which is extremely common in nature (73). The structural of polysaccharides is essential for their physiological functions. The anti-oxidative, anti-tumor, immune and other biological activities of polysaccharides are closely related to the monosaccharide composition, glycosidic bond and substituent types (74). Sulfated polysaccharides had anti-viral activity, but the activity of sulfated homopolysaccharides was greater than that of sulfated heteropolysaccharides, and the activity of β-1,3-D-glucan was significantly higher than that of β-1,6-D-glucan (75). *Atractylodes macrocephala Koidz* consists of 5 monosaccharides: rhamnose, glucose, mannose, xylose and galactose, with a molar ratio of 0.03:0.25:0.15:0.41:0.15. Its high xylose content enables the activation of proliferation of *Bifidobacterial* in the gut, which is beneficial to human health (76).

**Figure 2:**
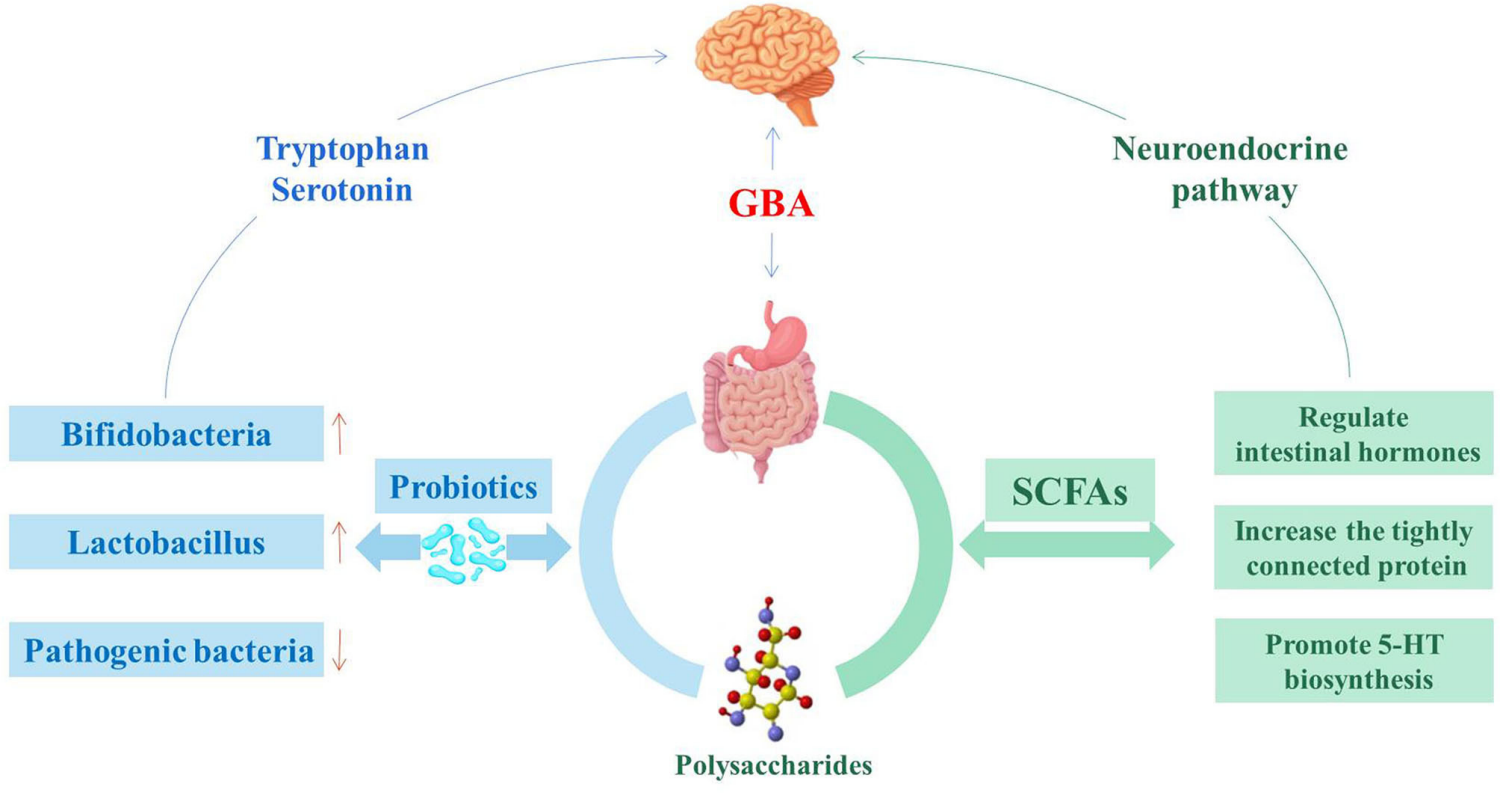
Probiotics and SCFAs are involved in intestinal flora regulation.

Polysaccharides have a variety of medicinal and nutritional benefits, including immunomodulation and gastrointestinal protection (77). The interaction between polysaccharides and intestinal flora has become a hot research topic, and the biological activity of polysaccharides has an important influence on the regulation of intestinal flora (Figure 2) (78). In addition, T lymphocytes, B lymphocytes, macrophages, and dendritic cells can be stimulated by polysaccharides or their gut metabolites and provide associated immune responses to maintain intestinal function and health (79). Chrysanthemum polysaccharides and glucose dietary supplements alone or in combination can increase the growth rate of *Penaeus vannamei* and the expression of related immune genes, enhance resistance to pathogens, and may activate the innate immune response by promoting the growth of beneficial bacteria in the gastrointestinal tract (80). Recently, studies have shown that polysaccharides can activate the immune system by stimulating the expression of anti-inflammatory cytokines, which are also currently used in innovative therapies (81).

IBS can be alleviated by non-medical treatment, such as changing surrounding conditions, regulating moods, and changing eating habits (82). Diet is a key determinant of gut microbial composition. Unhealthy diet can cause damage to the intestinal mucosal barrier, leading to the invasion or proliferation of harmful microorganisms, and triggering an inflammatory response (83). Researchers have found that if mice were switched from an unhealthy diet to a low fat, high fiber diet, their gut microbiota could change significantly within 1 day (84). Dietary interventions, such as polysaccharide administration, may become an effective therapeutic strategy to improve IBS (85–87). Polysaccharides can affect the intestinal microbial species, improving the abundance of beneficial bacteria, and reducing the abundance of pathogenic bacteria in the gut (88). During the treatment of intestinal flora with *Yupingfeng polysaccharides* (UYP) and *Yupingfeng polysaccharides* (FYP), the abundance of lactic acid bacteria increased significantly in the cecum and jejunum, while the abundance of *Enterococcus* decreased significantly in the cecum (89). A diet low in fermentable oligo-, di- and mono-saccharides, and polyols (FODMAP) can lead to changes in the gut microbiota and reduce symptoms of IBS, but the corresponding mechanism needs to be further studied (83).

In addition, the gut microbiota participates in the alleviation of IBS by producing metabolites such as SCFAs (90, 91). SCFAs are produced by bacterial fermentation in the colon, and involved in blood glucose metabolism, immune regulation and functions development, affect gastrointestinal peristalsis, and maintain the integrity of the intestinal wall barrier (92, 93). There are functional SCFAs receptors in the central and peripheral nervous systems. Propionic acid acts on the afferent nervous system around the portal vein by surrounding free fatty acid receptor 3, and then signals to the peripheral and central nervous system regions to induce intestinal gluconeogenesis (94). SCFAs can also affect the GBA through the secretion of hormones from the gut. SCFAs in the colon activate G-protein coupled receptors to stimulate enteroendocrine cells. This process may activate signaling cascades that affects the regulation of food intake in the brain circuits through systemic or vagal afferents (94).

## Conclusion

In this review, we focused on the modulatory effect of polysaccharides on intestinal flora and the implication for IBS from the perspective of GBA. Present studies have shown that polysaccharides are important for maintaining gastrointestinal homeostasis. However, it is not clear the relationship between the molecular weight, monosaccharide composition and glycosidic linkage of polysaccharides and their regulation of gut microbiota. In further studies, attentions should be paid to elucidate the relationship between IBS and intestinal flora, especially the GBA.

## Author Contributions

YY: conceptualization and writing original draft. YL and PZ: supervision and writing original draft. KC: supervision and validation. ZW: writing review and editing. XZ: supervision and writing review and editing. All authors contributed to the article and approved the submitted version.

## Funding

This work was sponsored by Zhejiang Provincial Key Research and Development Program (2020C02037) and People-benefit Project of Ningbo (202002N3078).

## Conflict of Interest

The authors declare that the research was conducted in the absence of any commercial or financial relationships that could be construed as a potential conflict of interest.

## Publisher's Note

All claims expressed in this article are solely those of the authors and do not necessarily represent those of their affiliated organizations, or those of the publisher, the editors and the reviewers. Any product that may be evaluated in this article, or claim that may be made by its manufacturer, is not guaranteed or endorsed by the publisher.
